# Screening of flavor-enhancing yeast and its role in fig fermentation

**DOI:** 10.3389/fbioe.2026.1807323

**Published:** 2026-05-12

**Authors:** Zhifei Chen, Yizhuo Shentu, Lei Li, Qingfu Wang, Yongzhen Zhao, Wenyuan Qi, Shen Huang, Yibo Ning, Qiuling Wang

**Affiliations:** 1 Technology Center for China Tobacco Henan Industrial Limited Company, Zhengzhou, Henan, China; 2 College of Tobacco Science and Engineering, Zhengzhou University of Light Industry, Zhengzhou, China

**Keywords:** aroma compounds, fermentation, FIG, T.delbrueckii, whole-genome sequencing

## Abstract

**Background:**

Figs (*Ficus carica* L.) are rich in sugars and bound aroma precursors that can be converted into aroma compounds.

**Methods:**

In this study, the W-8 strain was isolated from fig epidermis and identified as *Torulaspora delbrueckii* by 26S rDNA sequencing. By optimizing fermentation conditions using single-factor experiments and an orthogonal design, aroma compound (AC) production was evaluated. The whole genome of strain W-8 was sequenced and annotated.

**Results:**

AC production was 1.64 times that of the control. Isoamyl alcohol (0.264 mg/mL), phenethyl alcohol (0.053 mg/mL), isoamyl acetate (0.006 mg/mL), isobutyric acid (0.012 mg/mL), and 2-methylbutyric acid (0.004 mg/mL) were exclusively produced, and production of ethyl stearate and acetoin was increased by 4.20-fold and 2.30-fold, respectively. Strain W-8 harbors a complete genome of 9,224,546 bp. KEGG analysis showed prominent representation of amino acid and carbohydrate metabolism pathways, while GO annotation indicated enrichment in catalytic and metabolic processes. CAZy analysis identified key carbohydrate-active enzymes, including β-glucosidase.

**Conclusion:**

Genome analysis showed that *T. delbrueckii* W-8 possesses β-glucosidases and aroma-related metabolic pathways, providing a genetic basis for the release of bound aroma precursors from fig substrates and their conversion into AC. These findings highlight *T. delbrueckii* W-8 as a promising starter for enhancing aroma quality in fermented fig products.

## 1 Introduction

Natural aroma substances are increasingly favored by consumers compared with synthetic flavor substances and therefore drive the demand for the sustainable production of these compounds (Dzialo et al., 2017; Melini and Melini, 2024). Microbial fermentation has great potential for the production of various natural aroma compounds, including higher alcohols, esters, organic acids, and other volatile substances through various metabolic pathways, such as the Ehrlich pathway (Arshad et al., 2024; Hazelwood et al., 2008; Saerens et al., 2010). The precursors of these aroma compounds are found in many fruits in glycosidically bound forms (de Ovalle et al., 2021; Schober et al., 2023).

*Ficus carica* L. (fig) is rich in fermentable sugars, organic acids, phenolic acids, flavonoids, and other bioactive compounds (Arvaniti et al., 2019; Ayuso et al., 2022; Oliveira et al., 2010), making it a promising substrate for the production of aroma compounds through microbial fermentation. Microbial fermentation can break down the cell walls of figs, releasing more aroma compounds. Meanwhile, the metabolites produced by microorganisms themselves can increase the variety of aroma compounds in figs and enhance their aromatic quality.

Non-*Saccharomyces* yeasts have received extensive attention because of their ability to produce aroma compounds during the fermentation of different substrates (Padilla et al., 2016; Varela, 2016). *T*. *delbrueckii* produces low volatile acids, can significantly promote the release of aroma compounds, and generate floral, fruity, caramel, and other characteristic aromas (Benito, 2018; Cheng et al., 2025; Silva-Sousa et al., 2022). It is noteworthy that *T*. *delbrueckii* expresses β-glucosidase, which can hydrolyze glycoside-bound aroma precursors into free aroma compounds (de Ovalle et al., 2021; Schober et al., 2023). Thus, it is regarded as having potential application value. At the genomic level, recent studies have improved the genome annotation of *T*. *delbrueckii* and have shown that it is significantly enriched in metabolic functions related to fermentation performance (Santiago et al., 2021; Silva-Sousa et al., 2024).

However, at present, most research on *T. delbrueckii* focuses on wine and beer fermentation (Benito, 2018; Silva-Sousa et al., 2022; Zhang et al., 2018). Existing research on fig fermentation mostly uses *Saccharomyces cerevisiae* or lactic acid bacteria (Ma and Liu, 2010; Wang et al., 2023), and fermentation of figs using non-*Saccharomyces* yeasts for aroma production has not yet been reported.

In this study, a strain of *T. delbrueckii* (W-8) was isolated from fig surfaces and applied to produce aroma compounds through fig fermentation. The fermentation conditions were optimized using single-factor experiments and orthogonal design to achieve higher yield of aroma compounds. Therefore, the primary objective of this study is to utilize microbial fermentation as a biotechnological process to efficiently release and synthesize natural aroma compounds from figs. These volatile metabolites hold great potential as natural flavor extracts for the food and fragrance industries. Additionally, the whole genome of strain W-8 was sequenced and annotated using the KEGG, GO, and CAZy databases to predict key aroma-related enzymes and metabolic pathways.

## 2 Materials and methods

### 2.1 Screening of microorganisms from fig for enhancing aroma quality

Fresh figs used for yeast isolation were purchased from Dazhang Supermarket, Zhengzhou, China, while dried figs were obtained from Aksu Prefecture, Xinjiang, China. The epidermis of both fresh and dried figs was aseptically cut using a sterile scalpel, then fig tissue (10 g) was transferred into 90 mL of sterile water and mixed for 1 h at 30 °C and 180 rpm on a shaker (ZWYR-2102C, Shanghai Zhicheng). Subsequently, 5% (v/v) of the suspension was inoculated into YPD liquid medium and incubated at 28 °C and 150 rpm for 48 h in a shaking incubator. Afterwards, 100 μL of 10^−3^–10^−5^ dilutions were spread on YPD agar plates and incubated at 30 °C for 48 h in an incubator (DH-400, Beijing Zhongxing Weiye). The microbial colonies were examined under a light microscope. Colonies exhibiting typical yeast-like morphological features, such as oval or spherical cell shapes and signs of budding reproduction, were selected and cultured in YPD liquid medium. The isolates were streaked on fig solid medium (prepared by adding 2% w/v agar to the fig extract broth) for purification. For secondary screening, overnight cultures were inoculated (3%, v/v) into 30 mL fig fermentation medium (Powdered dried figs) prepared from 10 g dried figs per 100 mL water and incubated at 28 °C with shaking at 150 rpm for 36 h.

### 2.2 Sensory evaluation

Sensory evaluation was performed on the fermentation broth using fig as the fermentation substrate. Strains that significantly enhanced the aroma compounds of fig after fermentation were screened based on sensory evaluation scores. After fermentation, 10 mL of the fermentation broth was collected for odor evaluation. Quantitative descriptive analysis was conducted in accordance with GB/T 10220-2012 (Sensory analysis—Methodology—General guidelines), and eight trained sensory panelists with a background in food science evaluated the aroma attributes. The sensory analysis was carried out at room temperature (25 °C), and five attributes—floral, fruity, wine-like, fresh, and sweet—were assessed on an intensity scale ranging from 0 to 5. The final sensory scores were expressed as the mean values of the eight panelists.

### 2.3 DNA extraction and 26S rDNA sequencing of aroma-producing yeast

Genomic DNA from the W-8 strain was extracted from an overnight YPD culture by using the Yeast Genomic DNA Extraction Kit (Tiangen Biotech, Beijing, China). DNA quality and concentration were assessed using a NanoDrop spectrophotometer by measuring the A260/A280 ratio. The genomic DNA was submitted to Sangon Biotech (Shanghai) Co., Ltd. at 30 ng/μL for 26S rDNA sequencing. The D1/D2 domain of the 26S/28S rDNA gene was amplified using universal yeast primers: NL1: 5′-GCA​TAT​CAA​TAA​GCG​GAG​GAA​AAG-3′ and NL4: 5′-GGT​CCG​TGT​TTC​AAG​ACG​G-3′. The obtained sequences were compared with homologous sequences in the NCBI database using BLAST, and a phylogenetic tree was constructed in MEGA11.

### 2.4 Surface morphology of aroma-producing yeast

For SEM analysis, 1 mL of W-8 culture was harvested from the fermentation medium and centrifuged at 5,000 × g for 5 min. The pellet was washed with phosphate-buffered saline, fixed in 2.5% glutaraldehyde, and dehydrated through a graded ethanol series (30%–99%, 1 mL each step). The dehydrated cells were air-dried on aluminium stubs mounted with conductive adhesive, sputter-coated with a thin gold layer, and observed by SEM following previously described protocols (Chebotar et al., 2017; Watson et al., 1980).

### 2.5 GC–MS analysis of volatile compounds in fermented fig broth

In this study, the fermented fig broth without yeast inoculation was used as a blank control. To extract volatile compounds, the 10 mL fermentation broth was mixed with 15 mL dichloromethane, vortexed for 10 min, and centrifuged at 10,000 rpm for 5 min to separate the organic phase. The extraction was repeated three times. The combined organic extracts were dried overnight using anhydrous sodium sulphate and concentrated to 1 mL. The 2,6-dichlorotoluene (0.5211 mg/mL) was added to the concentrated extract and membrane-filtered (0.22 μm) before GC–MS analysis.

GC–MS analysis was performed using an Agilent 8890 GC coupled with a 5977B mass spectrometer equipped with a DB-5MS capillary column. The injector temperature was set at 280 °C, with a split ratio of 3:1. Helium was used as the carrier gas at a flow rate of 1.0 mL/min. The oven temperature program was as follows: initial temperature 50 °C held for 3 min, increased to 130 °C at 2 °C/min and held for 3 min, then raised to 180 °C at 5 °C/min and held for 2 min, and finally increased to 280 °C at 3 °C/min and held for 5 min. Mass spectrometry was conducted in electron impact (EI^+^) mode at 70 eV, with the ion source temperature maintained at 230 °C ± 1 °C and the quadrupole temperature at 150 °C. Data acquisition was initiated 7 min after injection to avoid solvent interference, and spectra were recorded in full scan mode (m/z 35–500). Volatile compounds were identified by comparison with the NIST 20 mass spectral library using a match threshold of ≥70%, and semi-quantification was performed using the 2,6-dichlorotoluene internal standard. The total content of aroma compounds was calculated using the internal standard method, defined as the ratio of the total peak area of all aroma compounds to the peak area of the internal standard, multiplied by the concentration of the internal standard, to yield the total aroma content (mg/mL).

### 2.6 Optimization of fermentation conditions for aroma-producing yeast strain

A large single colony of the W-8 strain was inoculated into YPD liquid medium and cultured for 36 h under the growth conditions mentioned above. Optical density at 600 nm was measured every 4 h, and a growth curve was constructed. The fermentation process parameters were optimized through single-factor experiments followed by an orthogonal experiment (Table 1). Fermentation time, fermentation temperature, and initial pH were selected as the main factors affecting aroma production. The relative content of aroma compounds (%)in the fermentation broth were used as the evaluation index. The relative content of aroma compounds (%) was calculated as the ratio of the total volatile compound concentration under a specific fermentation condition to the maximum total volatile compound concentration achieved among all tested conditions in that single-factor experiment.

**TABLE 1:** Factors and levels for the orthogonal experiment design.

Level	Factor		
	A fermentation time (h)	B fermentation temperature (°C)	C initial pH value
−1	24	28	6.0
0	36	30	7.0
1	48	32	8.0

Single-factor experiments were conducted to evaluate the effects of fermentation time, temperature, and initial pH. For fermentation time optimization, cultures were incubated at 30 °C, 150 rpm, and an initial pH of 5, and samples were collected at 12, 24, 36, 48, and 60 h. Based on the optimal fermentation time, the effect of temperature was examined by cultivating the strain at 25, 28, 30, 32, and 35 °C under the same pH and shaking conditions. Subsequently, the effect of initial pH was evaluated at the optimized time and temperature by adjusting the medium pH to 5, 6, 7, 8, and 9, while keeping all other parameters constant.

### 2.7 Whole-genome sequencing of *Torulaspora delbrueckii* W-8

#### 2.7.1 Sample preparation for sequencing

Strain W-8 was grown in YPD medium for 24 h, and genomic DNA was extracted using a yeast genomic DNA extraction kit (Tiangen Biotech, Beijing, China) according to the manufacturer’s instructions. DNA concentration and purity were assessed using a NanoDrop 2000 spectrophotometer (Thermo Fisher Scientific, United States) by measuring the A260/A280 ratio. The extracted genomic DNA was stored at −20 °C until sequencing. Whole-genome sequencing was performed by Sangon Biotech (Shanghai) Co., Ltd.

DNA extracted from *T. delbrueckii* W-8 was used to construct the library. Based on concentration and purity qualifications, 20 kb fragment libraries were constructed. *De novo* sequencing of the yeast genome was performed using second-generation Illumina HiSeq technology. Raw sequencing data were stored in FASTQ format, and quality control was performed to remove low-quality reads and adapters, yielding high-quality, clean data for downstream analysis.

#### 2.7.2 Gene prediction and functional annotation

Protein-coding genes in the fungal genome were predicted using GeneMark-ES/ET software (Lomsadze et al., 2014; Ter-Hovhannisyan et al., 2008). Sequence alignment of predicted protein-coding genes against the KEGG database (Kanehisa and Goto, 2000) was performed using Diamond software with an E-value cutoff of <1e−5 (Buchfink et al., 2014). Functional annotation against the GO database (The Gene Ontology Consortium, 2015) was conducted using BLASTP, with the sequence alignment threshold set to E-value ≤ 1e−5. Prediction of CAZy enzyme genes was performed using the hmmscan software (Eddy, 2011), selecting sequences with E-value <1e−5 for ORF lengths >80 amino acids, E-value <1e−3 for alignment lengths <80 amino acids, and where the alignment length exceeded 30% of the reference sequence length (Lombard et al., 2014).

## 3 Results and discussion

### 3.1 Screening of aroma-producing non-canonical yeast from fig

#### 3.1.1 Screening and sensory selection of candidate strains

Initially, 20 yeast isolates were obtained from the fig samples. During the secondary screening, these isolates were evaluated based on sensory scoring. Ultimately, three strains exhibiting the most pronounced aroma intensities were selected and purified for further analysis.

**FIGURE 1:**
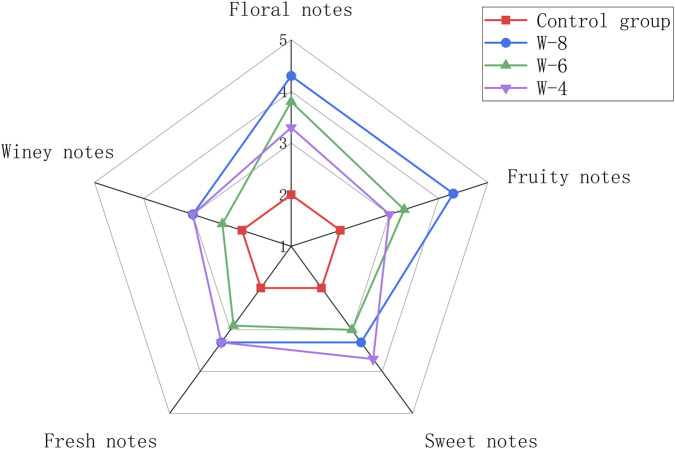
Odor evaluation radar chart of fig fermentation broth.

To systematically evaluate the impact of different strains on the sensory quality of the fermented product, quantitative descriptive analysis (QDA) was performed across five core aroma attributes: floral, fruity, sweet, fresh, and winey notes. The non-inoculated control group served as the sensory baseline, with all attributes uniformly scored at 2.0. As visualized in the radar chart (Figure 1), all tested strains significantly improved the overall sensory profile relative to the control, with distinct strain-specific patterns of aroma enhancement observed. Strain W-8 showed the strongest overall aroma performance, with scores of 4.3 for floral and fruity attributes, 3.3 for sweet and fresh attributes, and 3.0 for the winey attribute. Strain W-6 displayed moderate enhancement, with scores of 3.8, 3.3, 3.0, 2.9, and 2.4 for floral, fruity, sweet, fresh, and winey attributes, respectively. Strain W-4 also improved the aroma profile and showed a relatively stronger sweet attribute (3.7).

These sensory characteristics are typically associated with higher alcohols and ester compounds, such as phenethyl alcohol, isoamyl acetate, and ethyl esters, which are known to contribute floral and fruity aromas in fermented products (Yan et al., 2019). Similar sensory-guided screening strategies have been widely used to identify aroma-enhancing non-Saccharomyces yeasts from fruits and spontaneous fermentations (Liu et al., 2022; Zhang et al., 2018). Based on its superior aroma intensity and balanced sensory profile, strain W-8 was selected for subsequent sequencing and phylogenetic analysis.

### 3.2 Identification and characterization of a non-canonical yeast isolated from fig

#### 3.2.1 Morphological characterization

**FIGURE 2:**
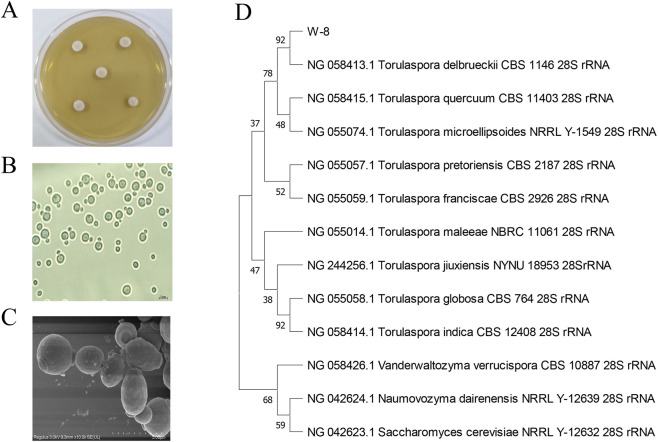
Morphological and phylogenetic characterization of strain W-8. Representative colony morphology of strain W-8 grown on solid medium is shown **(A)**; Light microscopy reveals individual yeast cells **(B)**, while scanning electron microscopy reveals an ellipsoidal cell shape **(C)**. The right panel presents a phylogenetic tree based on the 26S/28S rRNA genes **(D)**.

Strain W-8 was identified via a polyphasic approach combining morphological and molecular analyses. When cultured on YPD agar at 30 °C for 48–72 h, W-8 formed circular, creamy-white colonies with entire smooth margins and a slightly raised, rough surface (Figure 2A). Light microscopy revealed oval to ellipsoidal vegetative cells, occurring primarily as single cells or small clusters (Figure 2B), while SEM confirmed regular ellipsoidal cells with smooth walls and no hyphal/pseudohyphal structures (Figure 2C). These morphological traits align with the established phenotypic characteristics of the genus *Torulaspora* (Bastías et al., 2025; Januszek et al., 2020; Varela, 2016).

#### 3.2.2 Molecular identification based on 26S rDNA sequencing

Molecular identification of strain W-8 was performed by sequencing a 587 bp fragment of the 26S rDNA gene. BLAST analysis showed 100% sequence identity with *T. delbrueckii*. Consistent with this result, phylogenetic analysis placed strain W-8 firmly within the *T. delbrueckii* clade (Figure 2D), where it clustered closely with reference *T. delbrueckii* sequences. Together, these molecular analyses confirm that strain W-8 belongs to *Torulaspora delbrueckii* (Díaz-Montes and Castro-Muñoz, 2021; Serra et al., 2025).

#### 3.2.3 Growth characteristics of the aroma-producing yeast strain W-8

**FIGURE 3:**
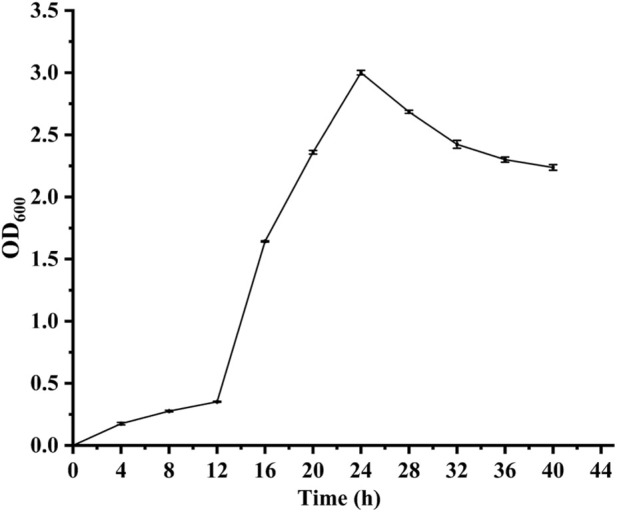
Time-course of microbial growth based on OD_600_ measurements.

The growth curve of strain W-8 exhibits a lag phase, an exponential growth phase, and a stationary phase at fermentation times of 0–12 h, 12–22 h, and 22 h, respectively (Figure 3). The lag phase likely reflects adaptation of strain W-8 to the fig medium, whereas the stationary phase was likely associated with nutrient depletion and reduced oxygen availability (Bisschops et al., 2015; Broach, 2012; Henriques et al., 2021; Zhang et al., 2018).

#### 3.2.4 Effects of fermentation parameters on aroma production by strain W-8

**FIGURE 4:**
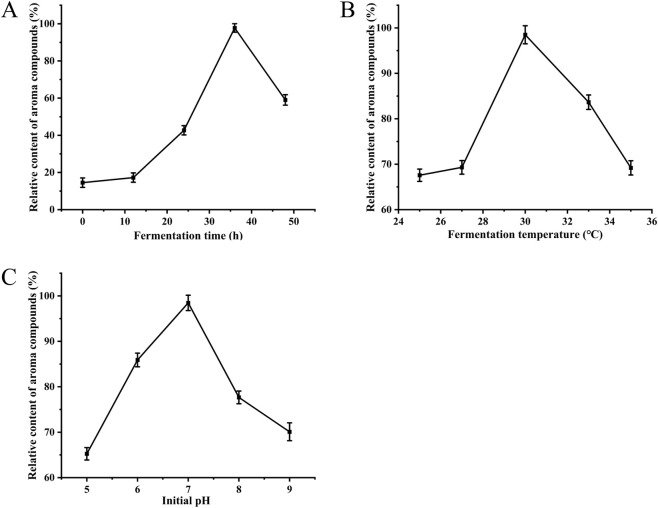
Effects of fermentation parameters on aroma compound production by strain W-8. **(A)** Effect of fermentation time on the relative content of aroma compounds. **(B)** Effect of fermentation temperature on aroma compound production. **(C)** Effect of initial pH on aroma compound production.

Figure 4A shows that the production of aroma compounds increased slowly during the first 12 h of fermentation, then increased rapidly to about 95% by the 36th hour; afterward, their production decreased to 58% over the next 48 h. The production of aroma compounds exhibited a trend of increasing initially and then decreasing. This pattern may be associated with the availability or depletion of nutrients and oxygen, as well as microbial growth metabolism at different fermentation stages in the fig medium. Specifically, the yield of aroma compounds increased sharply during the exponential growth phase of the microorganisms. However, as nutrients were heavily consumed and the microbes entered the stationary phase, the compound levels showed a downward trend. Additionally, other factors such as the volatility of aroma compounds and their utilization by microorganisms cannot be ruled out (Catrileo et al., 2020; Zhang et al., 2018). Figure 4B shows that aroma compound production was about 60% at 25 °C. When the W-8 strain was grown at 30 °C, production increased to 100%. However, further increases in temperature to 32 °C and 35 °C resulted in decreases to 83% and 69%, respectively. These results indicate that 30 °C is the optimal temperature for enzyme activity and cell growth, as yeast generally grows well at 30 °C (Sun et al., 2024). The results shown in Figure 4C indicate that aroma compound production increased as the pH increased from 5 (65%) to 7 (96%); however, at pH 8, production decreased to 78%. The pH of the medium influences cell growth and enzyme activity; therefore, it regulates the production of secondary metabolites in microorganisms (Caspeta et al., 2015; Thomas et al., 2002).

#### 3.2.5 Optimization of fermentation conditions for aroma production by strain W-8

Table 2 summarizes the parameters used in the orthogonal experiments and the corresponding production of aroma compounds by strain W-8. The production of total volatile aroma compounds ranged from 0.556 to 1.359 mg/mL under different fermentation conditions, reflecting the influence of fermentation parameters on aroma formation. The highest production level (1.359 mg/mL) was obtained under condition A_2_B_2_C_2_, corresponding to a fermentation time of 36 h, temperature of 30 °C, and pH 7. These results are consistent with those obtained from the single-factor experiments.

**TABLE 2:** Orthogonal experiment design and results for optimization of W-8 fermentation proces**s**.

Experiment no.	A fermentation time (h)	B fermentation temperature (°C)	C initial pH	Total volatile aroma compounds of W-8 (mg/mL)
1	24	28	6	0.55567
2	36	28	7	0.78435
3	48	28	8	0.67751
4	24	30	8	0.92026
5	36	30	7	1.35882
6	48	30	6	1.09489
7	24	32	7	0.92342
8	36	32	8	0.75732
9	48	32	6	0.58806
K_1_	2.40	2.02	2.24	​
K_2_	2.90	3.37	3.07	​
K_3_	2.36	2.27	2.36	​
k_1_	0.80	0.67	0.75	​
k_2_	0.97	1.12	1.02	​
k_3_	0.79	0.76	0.79	​
Range R	0.18	0.45	0.28	​

Variance analysis further evaluated the relative influence of these parameters (Table 3). The results indicate that fermentation temperature (factor B) significantly affected aroma compound production. The calculated F-value (11.29) was higher than F_0_._1_(2,2) = 9.0 but lower than F_0_._05_(2,2) = 19.0, indicating significance at the α = 0.10 level. In contrast, the variance analysis indicated that pH (C) and fermentation time did not significantly influence the production of aroma compounds.

**TABLE 3:** Variance analysis of orthogonal experiment result**s**.

Source of variation	Sum of squares	Degrees of freedom	Mean square	F-value
Fermentation time (A)	0.028	2	0.014	0.82
Fermentation temperature B	0.385	2	0.192	11.29
Initial pH C	0.156	2	0.078	4.59
Error	0.034	2	0.017	​
Total	0.603	8	​	​

F-values were evaluated at α = 0.10 and α = 0.05. indicates significance at α = 0.10, and indicates significance at α = 0.05.

Range (R) analysis further demonstrated that fermentation temperature exerted a greater influence on aroma compound production than pH and fermentation time. Accordingly, the importance of these factors can be ranked as B > C > A. Temperature is known to strongly influence microbial cell growth, membrane fluidity, and enzyme activity; consequently, it plays a critical role in regulating metabolic reactions involved in aroma compound formation (Canonico et al., 2020; Papadimitriou et al., 2016; Zhang et al., 2018; Zhang et al., 2024).

Based on the orthogonal experimental results, the optimal fermentation conditions for maximizing aroma compound production were determined to be A_2_B_2_C_2_ (36 h, 30 °C, and pH 7).

### 3.3 GC–MS analysis of volatile aroma compounds in W-8–fermented fig medium

Table 4 presents the GC–MS results, showing that the total aroma compound production in the fig medium fermented by W-8 increased by 1.64-fold (1.41928 mg/mL) compared with the non-fermented blank control (CK, 0.863 mg/mL).The production of several classes of compounds, including alcohols, esters, ketones, acids, and aldehydes, was altered in the W-8–fermented fig medium.

**TABLE 4:** Changes of volatile aroma compounds in W-8–fermented Fig Medium.

Category	Compound	Rt (min)	Characteristic m/z	RIcalc	RIlit	Match quality (%)	CK (mg/mL)	W-8 (mg/mL)	Change (fold)
Alcohols	Isoamyl alcohol	9.02	45, 55, 70	1002	998	98.73	—	0.26398	Appeared in W-8
	p-Menthane-1-ol	34.04	81, 95, 138	1356	1352	85.98	0.00172	0.00145	0.84× (↓)
	2-Methyl-3-pentanol	72.35	56, 69, 87	2085	2082	86.99	0.00556	0.00618	1.11× (↑)
	2-Hexyl-1-decanol	45.09	55, 69, 83	1620	1617	88.54	0.00637	0.00544	0.85× (↓)
	(2R,3R)-(−)-2,3-Butanediol	10.88	45, 57, 74	1045	1043	95.61	—	0.01390	Appeared in W-8
	2,3-Butanediol	11.34	45, 57, 74	1058	1056	91.09	—	0.00167	Appeared in W-8
	Phenethyl alcohol	32.41	91, 108, 122	1120	1118	98.70	—	0.05258	Appeared in W-8
	2-Butyl-1-octanol	28.95	55, 69, 83	1480	1478	88.46	0.00234	0.00233	1.00× (≈)
Esters	Isoamyl acetate	8.19	43, 60, 70	925	923	95.51	—	0.00632	Appeared in W-8
	Lup-20(29)-en-3-yl acetate	92.83	189, 218, 234	2580	2577	89.15	—	0.30787	Appeared in W-8
	Ethyl stearate	79.46	88, 101, 157	2210	2208	89.77	0.00652	0.02739	4.20× (↑)
	Ethyl palmitate	73.35	88, 101, 143	2105	2103	92.98	0.08531	0.07531	0.88× (↓)
	Ethyl oleate	78.73	55, 69, 83	2195	2193	92.47	0.19087	0.20359	1.07× (↑)
	Octadecyl acetate	79.89	43, 55, 69	2220	2217	90.93	0.00808	0.00870	1.08× (↑)
	3-Hydroxy-2-butanone	8.19	43, 45, 88	920	918	95.51	0.00926	0.02131	2.30× (↑)
Ketones	Isophorone	32.89	82, 95, 109	1135	1133	97.42	0.03657	0.03340	0.91× (↓)
	Isobutyric acid	10.20	43, 45, 73	1030	1028	90.54	—	0.01183	Appeared in W-8
Acids	Palmitic acid	72.68	60, 73, 129	2090	2088	95.38	0.44604	0.34481	0.77× (↓)
	2-Methylbutyric acid	10.50	41, 57, 74	1038	1036	90.00	—	0.00411	Appeared in W-8
	Stearaldehyde	76.70	57, 71, 82	2160	2158	84.79	0.04225	0.02516	0.60× (↓)
Aldehydes	Palmitaldehyde	70.79	57, 71, 82	2050	2048	95.33	0.02264	0.01553	0.69× (↓)
	3,4-Dimethylbenzaldehyde	39.86	77, 91, 134	1260	1258	98.62	0.01029	0.01194	1.16× (↑)
Total	Compound	—	—	—	—	—	0.86316	1.41928	1.64×(↑) = +64.4%

Strain W-8 produced several key alcohols, among which isoamyl alcohol (0.26398 mg/mL) and phenethyl alcohol (0.05258 mg/mL) were detected exclusively in the fermentation medium. Isoamyl alcohol contributes fruity and alcoholic notes, while phenethyl alcohol contributes a rose-like floral aroma. These compounds belong to microbial volatile organic compounds that are known to influence aroma characteristics in fermented systems (Hazelwood et al., 2008; Mitri et al., 2022; Nancolas et al., 2017; Shemshura et al., 2024). Mechanistically, the exclusive production of these higher alcohols is primarily driven by the Ehrlich pathway, where the yeast metabolizes specific amino acids (such as leucine for isoamyl alcohol and phenylalanine for phenethyl alcohol) through sequential transamination, decarboxylation, and reduction processes. This robust synthesis of volatile alcohols is consistent with findings in fruit fermentations, where T. delbrueckii is frequently reported to elevate fruity and floral ester precursors more efficiently than conventional yeast strains (Zhang et al., 2018).

In addition, acetoin (3-hydroxy-2-butanone), which contributes creamy and buttery sensory notes, increased from 0.00926 mg/mL to 0.02131 mg/mL in the W-8–fermented fig medium (Table 4).

The banana-like ester isoamyl acetate (0.00632 mg/mL) was detected only in the W-8–fermented medium (Table 4), indicating enhanced ester formation during fermentation. Ethyl stearate, ethyl oleate, and octadecyl acetate, which are known to improve mouthfeel, are commonly found in fermented products (Fukuda et al., 1998; Holt et al., 2019; Nancolas et al., 2017; Saerens et al., 2010; Verstrepen et al., 2003). Among these compounds, ethyl stearate increased by 4.20-fold (0.02739 mg/mL), whereas ethyl oleate and octadecyl acetate showed moderate increases (Table 4).

The production of isobutyric acid (0.01183 mg/mL) and 2-methylbutyric acid (0.00411 mg/mL) was detected only in the W-8–fermented medium (Table 4). At low concentrations, these compounds can enhance aroma complexity (Díaz-Maroto et al., 2005; Dzialo et al., 2017).

In contrast, the production of long-chain fatty acids, such as palmitic acid, decreased from 0.44604 to 0.34481 mg/mL (Table 4), a phenomenon commonly observed in aroma-producing strains (Bizzio et al., 2022). Similarly, the production of most aldehydes, including stearaldehyde and palmitaldehyde, also decreased. These reductions may be associated with a lower accumulation of compounds related to fatty off-notes (Bizzio et al., 2022; Delač Salopek et al., 2022; Hazelwood et al., 2008; Paramithiotis et al., 2025).

Overall, the changes in volatile compound composition are consistent with the enhanced floral, fruity, and sweet sensory characteristics observed in the W-8–fermented fig medium.

### 3.4 Genomic features and functional metabolic potential of W-8 strain

Whole-genome sequencing of *Torulaspora delbrueckii* W-8 revealed a genome size of 9224546 bp, consistent with previously reported genome sizes of non-Saccharomyces yeasts (Albertin and Marullo, 2012). The genome assembly showed good continuity, with an N50 of 836,209 bp, indicating a reliable assembly suitable for downstream functional analysis (Walker et al., 2014). The GC content was 40.69%. Compared to the model strain CBS1146 (genome size 9.52 Mb, GC content 41.9%), there are some minor differences (Fernandes et al., 2021); however, it remains comparable to that of other T. delbrueckii strains. Additionally, a total of 4,697 protein-coding genes were predicted (Table 5).

**TABLE 5:** Basic genomic characteristics of *Torulaspora delbrueckii* W-8.

Indicator	Total length (bp)	GC content (%)	N50	Number of CAZy annotated genes	Number of predicted genes
Results	9224546	40.69	836209	33	4697

**FIGURE 5:**
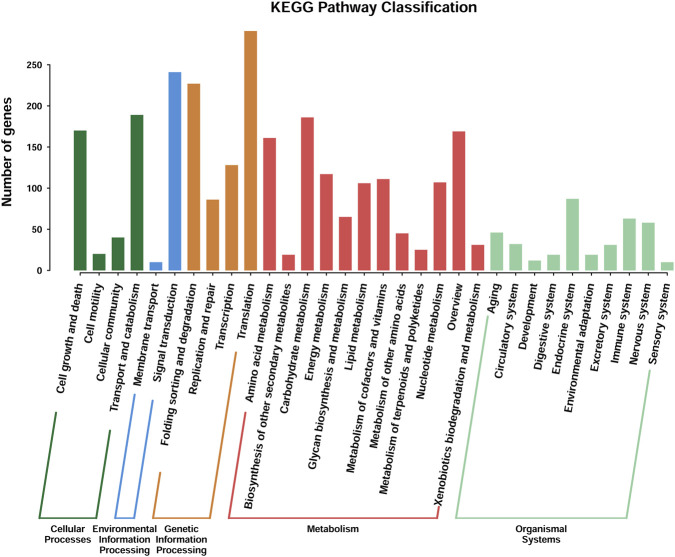
KEGG pathway classification of Torulaspora delbrueckii W-8 genome.

KEGG functional annotation assigned 4,050 genes to known biological pathways. As shown in Figure 5, metabolism represented the largest functional category (1,527 genes), reflecting the strong metabolic potential of this strain. Genetic information processing (826 genes), organismal systems (635 genes), and cellular processes (594 genes) were also well represented, suggesting robust transcriptional activity and effective cellular regulation during fermentation. In addition, 468 genes associated with environmental information processing indicate the capacity of strain W-8 to respond to environmental changes during fermentation.

Within the metabolism category, carbohydrate metabolism (287 genes) and amino acid metabolism (244 genes) were the most enriched subpathways. These pathways are closely linked to the formation of volatile compounds during fermentation, indicating a strong genetic capacity of the W-8 strain for converting carbohydrates and amino acids into aroma-related metabolites. Specifically, the high abundance of amino acid metabolism genes provides a robust genetic foundation for the aforementioned Ehrlich pathway. This genomic trait directly explains the underlying biological mechanism for the elevated levels of higher alcohols (e.g., isoamyl alcohol and phenethyl alcohol) observed in our GC-MS data. Furthermore, active carbohydrate metabolism ensures a continuous supply of carbon skeletons and energy (ATP) required for the subsequent synthesis of volatile esters.

Genes related to nucleotide metabolism (152 genes), energy metabolism (121 genes), and cofactor and vitamin metabolism (112 genes) further support active cellular growth and enzymatic activity during fermentation (Albertin and Marullo, 2012).

Additional genes involved in glycan biosynthesis and metabolism (80 genes) and xenobiotic biodegradation (53 genes) suggest the ability of strain W-8 to adapt to the complex biochemical environment of fig fermentation. A smaller set of genes associated with secondary metabolite biosynthesis (26 genes) and terpenoid and polyketide metabolism (25 genes) also indicates potential for producing diverse bioactive compounds contributing to aroma complexity (Canonico et al., 2020; Ciani et al., 2016; Varela, 2016).

The x-axis represents the specific secondary metabolic pathways, while the y-axis indicates the absolute number of genes assigned to each pathway. The pathways are grouped and color-coded into five major functional categories: Cellular Processes (dark green), Environmental Information Processing (blue), Genetic Information Processing (orange), Metabolism (red), and Organismal Systems (light green).

#### 3.4.1 GO functional annotation and its metabolic relevance in W-8 strain

**FIGURE 6:**
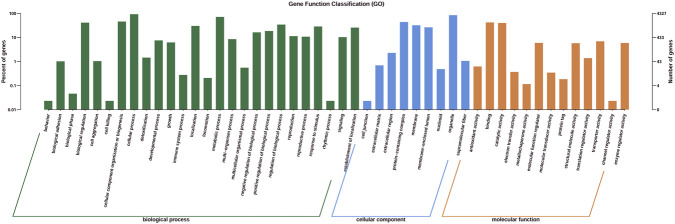
Gene ontology (GO) functional annotation of T. delbrueckii W-8.

Gene Ontology (GO) annotation of *T. delbrueckii* W-8 generated 334,317 annotations distributed across three GO domains-biological process, cellular component, and molecular function-covering 48 functional subclasses (Figure 6; Table 6). The biological process category accounted for the largest proportion of annotations, indicating active metabolic and biosynthetic processes in strain W-8 (Ciani et al., 2016; Santiago et al., 2021; Silva-Sousa et al., 2024; Tocci et al., 2023). This genetic enrichment provides a theoretical metabolic foundation that supports the diverse volatile metabolite production observed in the GC-MS analysis, which revealed diverse metabolites in the W-8–fermented fig medium (Kim et al., 2021). The cellular component category highlighted numerous membrane-associated proteins involved in nutrient transport, ethanol tolerance, and volatile compound secretion (Ma and Liu, 2010). Although the molecular function category contained fewer annotations, many genes encoding catalytic and binding proteins were identified. These included oxidoreductases, transferases, and hydrolases, suggesting that strain W-8 possesses strong enzymatic potential for biochemical transformations during fermentation (Pinu et al., 2017; Styger et al., 2011).

**TABLE 6:** GO annotation across three primary categorie**s**.

Categories	Gene counts
Biological process	224809
Cellular component	71211
Molecular function	38297

The annotated genes are classified into three primary GO domains, indicated by distinct colors along the x-axis: biological process (dark green), cellular component (blue), and molecular function (orange). The left y-axis shows the percentage of annotated genes, while the right y-axis indicates the absolute number of genes assigned to each specific functional sub-category.

#### 3.4.2 CAZy database annotation of W-8 strain

CAZy annotation indicated that strain W-8 encodes 33 carbohydrate-active enzyme genes distributed across four CAZy classes: AA (11), CE (3), GH (8), and GT (11) (Table 7). The GH enzyme set includes several activities related to carbohydrate utilization in fig substrates. For example, invertase (EC 3.2.1.26) catalyzes the hydrolysis of sucrose into glucose and fructose, thereby increasing the availability of fermentable sugars during early growth (Manoochehri et al., 2020). Similarly, enzymes such as α-amylase, pullulanase, glucoamylase, α-glucosidase, and α-1,6-glucosidase suggest the capacity of strain W-8 to hydrolyze starch, dextrins, and branched glucans into assimilable sugars (Far et al., 2020). The presence of endo-inulinase further indicates the potential utilization of fructan-type carbohydrates, reflecting metabolic versatility commonly observed in non-*Saccharomyces* fermentations (Cantarel et al., 2009).

**TABLE 7:** Analysis of CAZy enzyme gene clusters in strain W-8.

Enzyme	Family	Gene ID
α-Amylase (EC 3.2.1.1)	GH	ctg00012_0004316_t
Pullulanase (EC 3.2.1.41)	GH	ctg00012_0004316_t
Invertase (EC 3.2.1.26)	GH	ctg00016_0004534_t; ctg00003_0001668_t
Endo-inulinase (EC 3.2.1.7)	GH	ctg00016_0004534_t; ctg00003_0001668_t
Glucan 1,3-β-glucosidase (EC 3.2.1.58)	GH	ctg00007_0003034_t
	GH	ctg00008_0003567_t
Exo-β-1,4-glucanase (EC 3.2.1.74)	GH	ctg00008_0003567_t
Glucan endo-1,6-β-glucosidase (EC 3.2.1.75)	GH	ctg00008_0003567_t
Endo-β-1,4-glucanase (EC 3.2.1.4)	GH	ctg00008_0003567_t
α-Mannosidase (EC 3.2.1.113)	GH	ctg00001_0000022_t; ctg00001_0000047_t
β-Glucosidase (EC 3.2.1.21)	GH	ctg00008_0003567_t
Glucoamylase (EC 3.2.1.3)	GH	ctg00004_0001849_t
α-Glucosidase (EC 3.2.1.20)	GH	ctg00012_0004316_t
Starch α-1,6-glucosidase (EC 3.2.1.33)	GH	ctg00012_0004316_t

A particularly important enzyme identified in this study is β-glucosidase (EC 3.2.1.21) (Table 7). In fruits such as fig, many aroma-related terpenes and phenolic compounds occur in glycosidically bound, non-volatile forms. β-Glucosidase can hydrolyze these bonds and release free volatile aroma compounds (Schober et al., 2023). This observation strongly aligns with previous genomic and transcriptomic studies on wine strains of T. delbrueckii, which highlight its intrinsic β-glucosidase activity as a critical driver for unraveling bound aroma precursors and enhancing the floral complexity of fermented fruit substrates (de Ovalle et al., 2021; Silva-Sousa et al., 2024). In addition, GT enzymes are involved in glycosylation processes that contribute to cellular stability and stress tolerance during fermentation (Cantarel et al., 2009).

CAZy annotation results also highlight the potential of strain W-8 to release bound aroma precursors and enhance aroma compound formation during fermentation.

## 4 Conclusion

Natural aroma compounds are increasingly preferred over synthetic ones for several reasons, particularly their perceived health benefits and higher product quality. Keeping this in mind, this study aimed to use a non-*Saccharomyces* yeast for the production of aroma compounds using figs as the fermentation substrate. *T. delbrueckii* W-8 was isolated from figs and applied to fig fermentation under optimized culture conditions. As a result, *T. delbrueckii* W-8 enhanced the production of aroma compounds in fermented figs by 1.64-fold. The strain increased the production of several aroma compounds, including isoamyl alcohol, phenethyl alcohol, isoamyl acetate, isobutyric acid, and 2-methylbutyric acid, as well as elevated levels of ethyl stearate and acetoin during fermentation, indicating the broad substrate conversion potential of this strain. KEGG, GO, and CAZy analyses further revealed that *T. delbrueckii* W-8 possesses genetic potential for carbohydrate metabolism and enzymatic activities that may contribute to aroma compound formation during fermentation. The findings also suggest that similar bioconversion strategies could be applied to other fruit substrates to improve their flavor complexity and nutritional value. Although the current study successfully demonstrated the potential of the native strain W-8 in enhancing fig aroma, it should be noted that a commercial reference yeast (such as *S. cerevisiae*) was not included as a positive control. Future studies will incorporate comparative fermentation trials with standard commercial strains to further evaluate the competitive advantages of W-8 in industrial applications.

## Data Availability

The original contributions presented in the study are publicly available. This data can be found in the NCBI GenBank database under accession number PX915611. The data can be accessed via https://www.ncbi.nlm.nih.gov/nuccore/PX915611.
